# High-dimensional intravital microscopy reveals major changes in splenic immune system during postnatal development

**DOI:** 10.3389/fimmu.2022.1002919

**Published:** 2022-12-01

**Authors:** Maria Luiza Mundim Porto-Pedrosa, Camila Dutra Moreira de Miranda, Mateus Eustáquio Lopes, Brenda Naemi Nakagaki, Kassiana Mafra, Cristina Maria Pinto de Paula, Ariane Barros Diniz, Karen Marques de Oliveira Costa, Maisa Mota Antunes, André Gustavo Oliveira, Robert Balderas, Rodrigo Pestana Lopes, Gustavo Batista Menezes

**Affiliations:** ^1^ Center for Gastrointestinal Biology, Departamento de Morfologia, Instituto de Ciências Biológicas, Universidade Federal de Minas Gerais, Belo Horizonte, Minas Gerais, Brazil; ^2^ Departamento de Fisiologia, Instituto de Ciências Biológicas, Universidade Federal de Minas Gerais, Belo Horizonte, Minas Gerais, Brazil; ^3^ BD Biosciences, Department of Biological Sciences, San Jose, CA, United States; ^4^ BD Biosciences, Department of Medical & Scientific Affairs, São Paulo, São Paulo, Brazil

**Keywords:** spleen, malaria, development, immunology, *in vivo* imaging

## Abstract

Spleen is a key organ for immunologic surveillance, acting as a firewall for antigens and parasites that spread through the blood. However, how spleen leukocytes evolve across the developmental phase, and how they spatially organize and interact *in vivo* is still poorly understood. Using a novel combination of selected antibodies and fluorophores to image in vivo the spleen immune environment, we described for the first time the dynamics of immune development across postnatal period. We found that spleens from adults and infants had similar numbers and arrangement of lymphoid cells. In contrast, splenic immune environment in newborns is sharply different from adults in almost all parameters analysed. Using this in vivo approach, B cells were the most frequent subtype throughout the development. Also, we revealed how infections – using a model of malaria - can change the spleen immune profile in adults and infants, which could become the key to understanding different severity grades of infection. Our new imaging solutions can be extremely useful for different groups in all areas of biological investigation, paving a way for new intravital approaches and advances.

## 1 Introduction

The spleen is a unique secondary lymphoid organ that presents many important functions in immunity and blood homeostasis (1). Located on the left side of the abdominal cavity in mammals, it is the only lymphoid organ interposed in the circulatory system, which means that antigens reach the organ *via* blood, not through efferent lymphatic vessels. Blood comes from the splenic artery, flows through the arterioles of the white pulp (WP), and is delivered into cords in the red pulp (RP). After percolating the red pulp into an open blood system, blood is recollected in sinuses, enters the splenic vein, and returns to the circulatory system (2–4). Splenic architecture and its multicellular composition allow for blood surveillance and facilitate interactions between antigen-presenting cells (APCs) and lymphocytes, integrating innate and adaptive immune responses in an organized way (5). Besides, the spleen is responsible for destruction of senescent and damaged erythrocytes, and acts as a phagocytic “filter” (3). It also provides a site for extramedullary haematopoiesis in response to injuries (6) and plays a part as a reservoir of monocytes (7) and precursors of myeloid cells (4).

The spleen plays an important role in immune responses against blood-borne pathogens and parasitic infections, such as malaria. Malaria is a vector-borne parasitic disease caused by *Plasmodium* species and remains a major public health issue in tropical and subtropical regions worldwide (8). In 2020, there were an estimated 241 million cases and 627,000 deaths across the world (9). Children under 5 years of age are the most affected in population, representing approximately 60% of deaths in the globe (10). The parasite in question induces pronounced and varied splenic responses (11), modifying the organ’s size, architecture, and cellular composition (12). Therefore, further studies are required to better understand the pathophysiology of the disease in young subjects, as well as the advance of new tools that could potentially reduce the number of deaths.

In mice, the primordial spleen is detectable at approximately embryonic day E10.5-11 (13), and hematopoietic stem cells (HSC) and progenitor cells are found in the foetal spleen at embryonic day E13 (14). In fact, the spleen harbours a population of precursor cells derived from foetal liver (15) and its development is not fully completed at birth (16). And, regardless of its importance, how the spleen develops in postnatal phase is still a poorly understood subject. The splenic structure is organized into red and white pulp. Between these two regions there is a third area called the marginal zone (MZ), in rodents, or perifollicular zone, in humans (17). Each of these regions present different and specific leukocytes, as well as distinct functions and structures, respecting a very well-planned architecture (18). The red pulp is mostly constituted by macrophages, dendritic cells (DCs), and plasma cells. Marginal zone comprises two different subsets of macrophages, different populations of DCs and a distinct type of B cell. The white pulp is mainly lymphoid tissue, arranged in T and B cell zones, and contains most cells within the organ.

Despite the relevance of the geographical distribution of immune cells, their physical and dynamic relationship within the spleen, most studies have employed conventional histological techniques or flow cytometry to understand spleen biology. Limitations on these two main approaches may include: *i)* static view of an extremely dynamic environment imposed by classic histology; *ii)* loss of cell-cell interaction and cell location within the organ due to the tissue digestion procedures necessary for flow cytometry; *iii)* limitations of fluorescent markers in conventional immunofluorescence techniques, precluding precise immunophenotyping in spleen samples. In fact, while flow cytometry allows the quantitative analysis of cell populations, cell clustering during analysis is only based on similarity, but not in location or putative interaction. In contrast, microscopy methods provide spatial information (19), but structural visualization and quantification defined by multiple fluorophore combinations is still a major challenge (20). The demand for the development of new fluorophores that allow high dimensional immunophenotyping in flow cytometry - including up to 50 simultaneous parameters in a single sample - has created a plethora of molecule options in the market. The new generations of polymers (i.e. Brilliant Violet dyes; BV) allows excitation of multiple molecules using a single laser line (i.e. 405nm), but with emission in different spectra (ranging from 450 to > 780nm) (21). This allows the combination of multiple BV dyes with other conventional fluorophores, enhancing the number of parameters analysed simultaneously.

Therefore, these new synthetic fluorochromes have catalysed a revolution in the study of distinct cell populations enabling acquisition of more reliable data and description of novel cellular phenomena (22). On the other hand, intravital confocal microscopy is an advanced technique that has become widely used to visualize tissues and cells at high resolution in their native environment (23). However, due to inherent technical limitations or unfamiliarity of dye applications, most of the studies image only two or three channels, limiting the opportunities of cell identification and study *in vivo*. Here we describe a new panel of fluorescent dyes that, once conjugated to a selected list of antibodies, allowed an unprecedented visualization of the immune system *in vivo*, revealing new features of the development of the splenic immune system in the postnatal period, and during acute parasitic infections.

## 2 Material and methods

### 2.1 Animals

C57BL/6 mice were purchased from Centro de Bioterismo located at Universidade Federal de Minas Gerais (CEBIO–UFMG, Brazil) and bred in colonies. All animals were kept in microisolator acrylic cages (Alesco, Monte Mor, São Paulo, Brazil; three mice/cage) under controlled conditions of temperature (23°C). Water and autoclaved mouse food were given ad libitum and a dark/light cycle (12/12 h) was digitally monitored. All experiments with mice were approved by the Animal Ethics Committee from Universidade Federal de Minas Gerais (register number 143/2022), following international guidelines for animal care.

#### 2.1.1 Intravital imaging

Confocal microscopy imaging was performed as described previously (24). Mice were anesthetized (s.c.) with ketamine and xylazine (Syntec, 60 mg/kg and 15 mg/kg, respectively). Before surgery mice received a single dose of IgG (12,5 µg/g, Sigma-Aldrich) and after fifteen minutes they received a mixture of the following antibodies or fluorescent probes: anti-aCD31 BV421 (0,15 µg/g, clone 390, BD), anti-F4/80 BV480 (0,05 µg/g, clone T45-2342, BD), anti-Ly6C BV605 (0,1 µg/g, clone AL-21, BD), anti-aLy6G BV711(0,1 µg/g, clone 1A8, BD), anti-CD19 BB515 (0,1 µg/g, clone 1D3, BD), anti-NK1.1 PE (0,06 µg/g, clone PK136, BD) and anti-CD3e APC (0,06 µg/g, clone 145-2C11, BD) - all from BD Biosciences. In neonatal mice, delivery of drugs (via eye venous plexus) and surgery were made with minor modifications. All images were acquired using an inverted Nikon Eclipse Ti coupled to an A1R confocal microscope loaded with a spectral detector and XYZ motorized stage. Different fields of the organ were pictured. Blood flow analyses was done after FITC-albumin injection (12,5 µg/g, Sigma-Aldrich; i.v.) using resonant scanner (no time lapse). Images and videos rendering were made using NIS-Elements AR Analysis 5.41.01(Nikon) and Volocity(6.3) (Perkin Elmer, Waltham, MA).

#### 2.1.2 Infection with plasmodium

Frozen stocks of the *Plasmodium chabaudi* (AS) strain were thawed at room temperature and maintained into C57BL/6 mice for up to 10 passages (100μL/animal; i.p.). For experimental infection, infected red blood cells (iRBCs) were collected from donors and 1 × 10^5^ iRBCs injected intravenously into naive mice at two different ages: 2 and 7 weeks. These mice were observed daily and parasitemia was monitored by counting blood smears stained with rapid panoptic (Laborclin). One week after inoculation, some of the infected animals were submitted to confocal microscopy imaging. Survival rates of infected animals were assessed for thirty days after infection.

### 2.2 Image analysis and cell tracking

For each animal, three images were selected and submitted to cell counting. Each image was cropped and enlarged, allowing a reliable cell analysis and quantification of each field. This analysis was done manually using ImageJ. The average amount of cell types from the three images, for each animal of each group was plotted on the graphs. Cellular tracking, morphologic analysis and area measurement were performed semi automatically utilizing NIS Elements AR Analysis 5.41.01 (Nikon). Movies were recorded taking one frame every 30s. Tracking analysis was performed using only one animal.

#### 2.2.1 Statistical analyses

Experimental data were analysed using Prism 6.0 software (GraphPad Softwares, Inc., La Jolla, CA, USA). Data collected from assays with three test groups were assessed by analysis of variance (one-way ANOVA) and Dunnet’s *post hoc* test. Comparisons between two treatment groups (infected and non-infected animals) were evaluated by unpaired Student’s t test. *In vivo* experimental groups had at least five mice per group. All data are given as the mean ± SEM, and differences were considered statistically significant at p <0.05.

## 3 Results

### 3.1 Novel microscope imaging setup and custom-made fluorophores allow a simultaneous 7 channel intravital imaging acquisition

**Figure 1:**
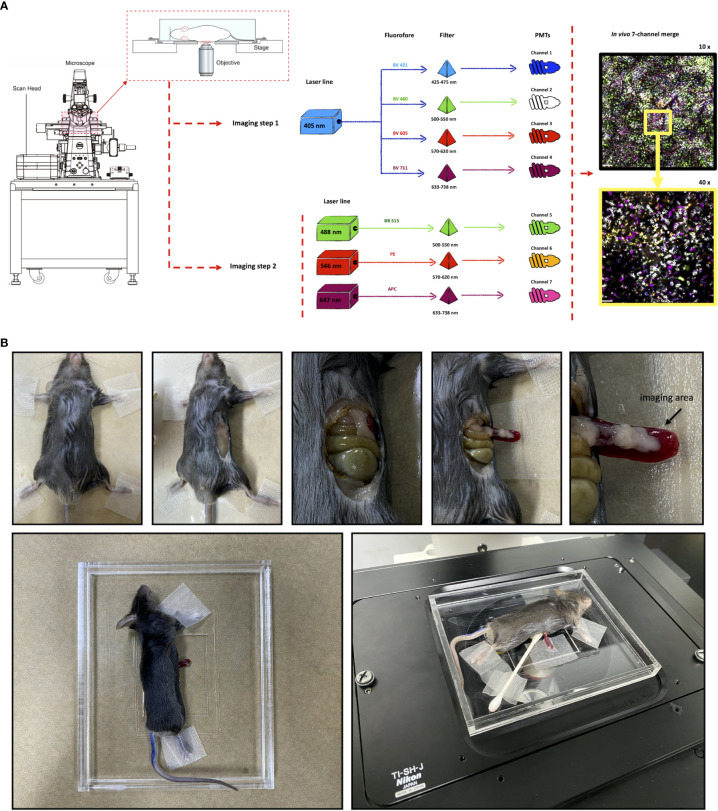
Novel microscope imaging setup and selected fluorophores allow seven channel intravital imaging acquisition. **(A)** Schematic diagram summarizing the new protocol used to identify a wide range of immune cells *in vivo*. **(B)** Detailed surgical procedure for spleen imaging using confocal intravital microscopy.

Precise *in vivo* immunophenotyping using conventional confocal microscopes can be very challenging. This is mainly due to limitations on availability on both laser lines and fluorophores that do not overlap the excitation/emission spectra. In general, stock microscopes are loaded with 3 or 4 lasers/filter/detectors sets, allowing the acquisition of images in 3 or 4 channels. In addition, commercially available fluorophores usually operate in a one-laser/one-filter fashion, which in turn limits imaging acquisition in 4 channels at maximum. To supersede these barriers and expand our capacity of multi-channel image acquisition using intravital microscopy, we developed a novel approach based on programming a conventional confocal microscope to perform advanced acquisition settings, together with a novel panel of custom-made antibodies. These antibodies were conjugated with new fluorophores, allowing 7 simultaneous excitation/emission strategies with virtually no spectral overlap. First, we developed an acquisition setup in the microscope based on a two-step imaging acquisition protocol, followed by an automatic 7 channel merge (**Figure 1**). The first imaging setup was done using a common setup where 3 fluorophores were excited and collected using regular settings. In this case, we used BB515, a FITC-similar fluorophore (excitation: 488nm laser, acquisition: 500-550 nm filter set), phycoerythrin (PE; excitation: 561nm laser, acquisition: 570-620 nm filter set) and allophycocyanin (APC; excitation: 488nm laser, acquisition: 633/738 nm filter set). Then, a second imaging protocol was dedicated to exciting fluorophores in the 405 nm range, which include all Brilliant Violet-conjugated antibodies. In this way, the other laser lines were not active, and we set up all 4 detectors to collect signal from BV421 (acquisition: 425-475 nm filter set), BV480 (acquisition: 500-550 nm filter set), BV605 (acquisition: 570-620 nm filter set) and BV711 (acquisition: 633-738nm filter set). Despite being excited by the same laser wavelength, their emission spectra are very divergent, with no significant spectral overlap. Combining these two setups, 7 simultaneous channels could be simultaneously imaged and merged.

### 3.2 Strategic conjugation of antibodies targeted to cell-surface receptors allowed an unprecedented identification of a wide range of immune cells *in vivo*

**Figure 2:**
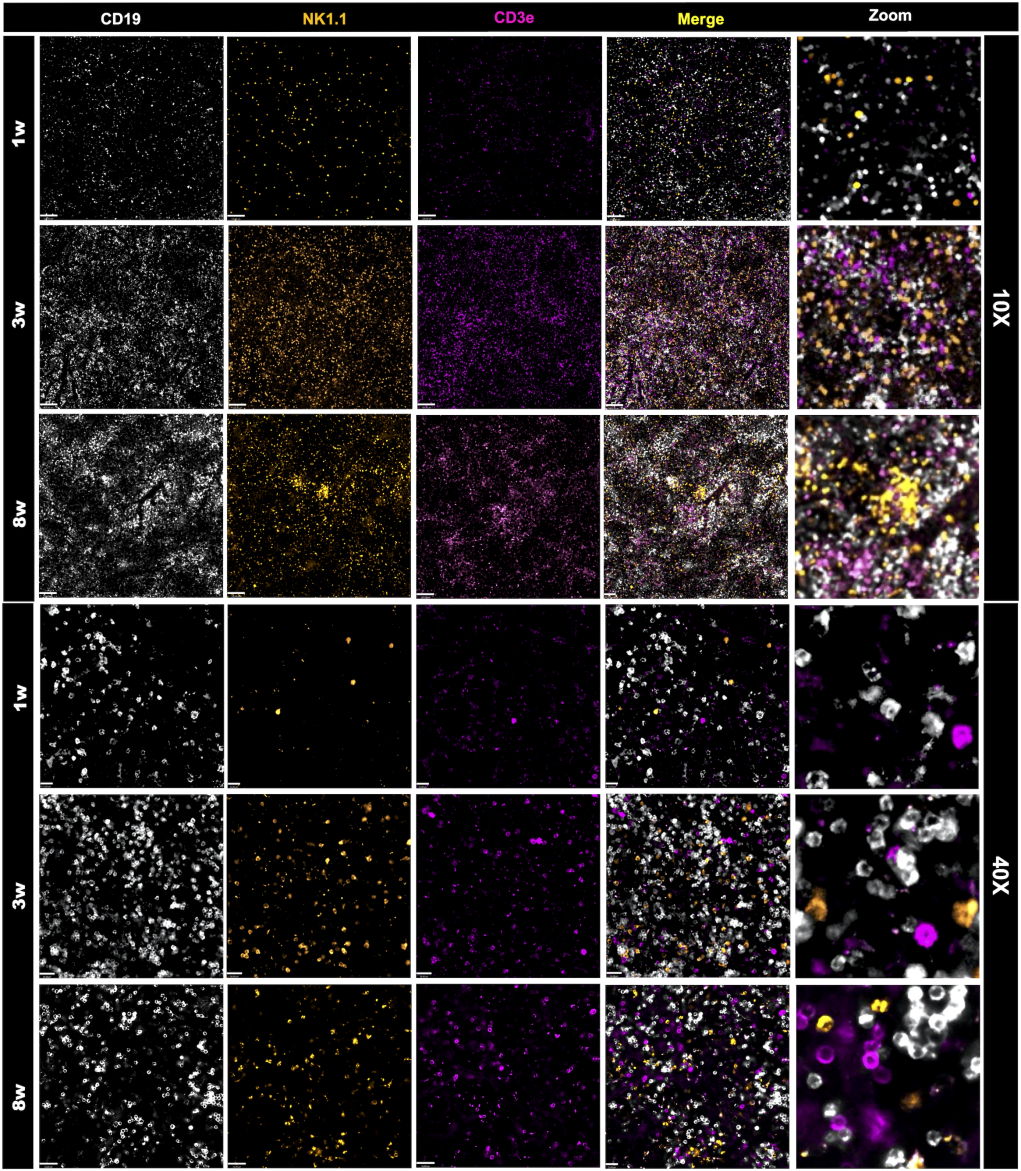
Changes in the frequency and organization of the lymphoid splenic compartment. Spleen staining in different ages using WT mice that received an intravenous injection containing anti-CD19 (in white), anti-NK1.1 (in orange) and anti-CD3e (in magenta). Scale bars = 120μm (10x); 21 μm (40x). Zoom images were enlarged 8 times.

**Figure 3:**
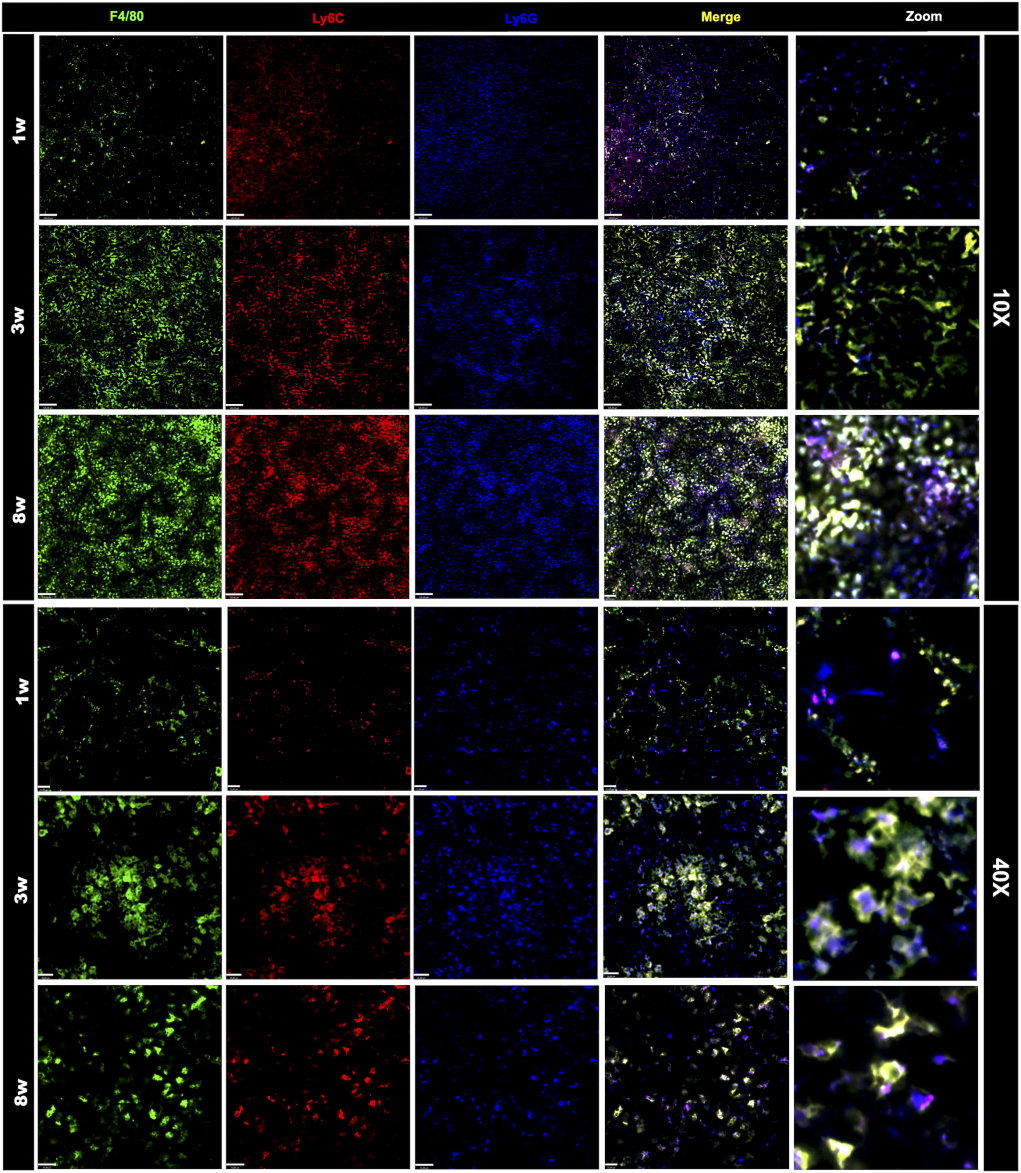
Changes in the frequency and organization of the myeloid splenic compartment. Spleen staining in different ages using WT mice that received an intravenous injection with anti F4-80 (in green), anti-Ly6C (in red), anti-Ly6G (in blue). Scale bars = 120μm (10x); 21 μm (40x). Zoom images were enlarged 8 times.

Once we have designed the optic strategy for image acquisition, we next planned an antibody panel that was directed only for cell-surface markers, allowing *in vivo* imaging of immune cells. For this, we selected target antigens that in combination would identify main leukocytes subsets, validating our *in vivo* strategy. Initially, all antibodies were tested individually to assure affinity and relative specificity, and since we used 7 different channels, we also started merging markers belonging to either lymphoid or myeloid compartments. We employed anti-CD19 to stain for B cells, anti-CD3 to broadly identify T cells (or CD3 expressing NKT cells), NK 1.1 to identify NK cells (NK 1.1+ CD3-) and NKT cells (NK 1.1+ CD3+). As shown in **Figure 2**, all three markers were successfully visualized, allowing the visualization of the large population of splenic lymphoid cells in all ages studied. We next setup to image splenic myeloid cells (**Figure 3**) using the same intravital microscopy. For this we selected markers that allowed the identification of macrophages (star-shaped F4/80+ Ly6C+ cells), neutrophils (round F4/80-, Ly6G+ cells) and monocytes (round Ly6C+ F4/80+ Ly6G-/low).

**Figure 4:**
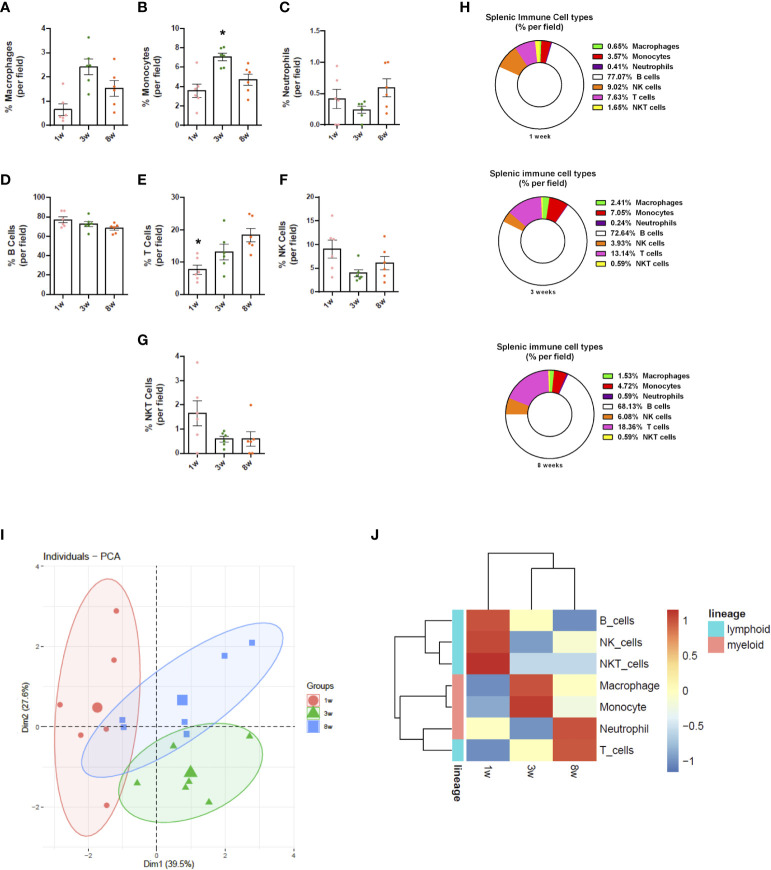
Splenic immune cell composition changes throughout life. Images from spleen confocal microscopy were manually quantified (n = 6/group; 3 fields per animal). **(A-H)** Differential expression of markers and cell morphology were analysed to define immune cell populations, which were quantified in frequency (% of total). **(I)** PCA analyses plot of the data obtained from the three groups evaluated. **(J)** Heatmap representation of the chronological dynamics of immune populations, clustered by cell-lineages. *p <0.05 compared to the adult mouse group (8 weeks). One-way ANOVA. NK, natural killer.

Due to limitations on channel availability, dendritic cells and eosinophils were not identified in these initial procedures; however, using dedicated cell surface markers, their imaging *in vivo* is also possible. In comparison to adults, spleens from newborns have significantly less myeloid cells. Macrophages were widely distributed within the spleen, and their spatial distribution resembled long cords of cells surrounding larger vessels. These cells also frequently presented vacuole inside cytoplasm, which also added in their identification as macrophages. Again, the macrophage population in newborns was significantly smaller in comparison to infants (3-fold smaller), and less frequent than adults (>50% reduction; **Figure 4A**). In contrast to lymphoid cells and other myeloid subtypes, spleen macrophages are star-shaped cells that seem to enhance F4/80 expression across the development. When we stained for Ly6C, an interesting scenario was observed. Most star-shaped cells were also positive for Ly6C, and were identified as macrophages, while round cells that were double-positive for F4/80 and Ly6C were considered as monocytes. Therefore, intravital imaging can add a new layer on the immunophenotyping process since not only surface markers, but also cell shape can be useful for these purposes. Despite fluctuations in infant phase, monocyte population in newborns (~3.5%) was also smaller in comparison to infants and adults (~7.0 and 4.7%, respectively; **Figure 4B**). Finally, staining for Ly6G revealed that a large part of tissue resident cells was double-positive for Ly6C and F4/80, and these cells - when round - were also considered as monocytes. However, we could identify a population of Ly6G+ Ly6C- F4/80- cells that were also round and were trafficking inside spleen vessels. These cells were considered as neutrophils and their frequency was stable across postnatal development (**Figure 4C**).

In agreement to myeloid cells, newborn spleens (1 week) harbour significantly less lymphoid cells compared to adults (8 weeks) and infants (3 weeks). These cells were also more distant from each other in neonates. Spleens from adults and infants had similar numbers and arrangement of lymphoid cells. Using this *in vivo* approach, B cells were the most frequent subtype throughout the development, comprising around 77% of all cells (lymphoid and myeloid) in all ages studied (**Figure 4D, H**). In fact, during postnatal development, B cells not only increased their number, but they also started to organize themselves into follicles, mainly in the neighbourhood of larger vessels. Also, probably due to their high frequency and numbers, B cells were frequently seen as doublets or large aggregates, showing that they might interact in a splenic B cell network *in vivo*. Also, their massive presence in all ages that we imaged suggest that these cells might play a key role in immune response not only in adults, but also in the first phases of neonatal development. When we stained for NK 1.1, we also observed a high number of NK 1.1+ cells, which were in turn more widely distributed across the spleen. Again, newborns displayed a smaller number of these cells, and a similar pattern was seen in both infants and adults. To add in our protocol for immune cell phenotyping, we counterstained *in vivo* with anti-CD3, allowing distinction of T cells, NK and NKT cells. As expected, CD3+ cells are also widely present in spleens, being visualized in every field imaged. Interestingly, T cell frequency in newborns was significantly lower in comparison to infants and adults. Using intravital microscopy, while neonates presented only ~7% of T cells within the spleen, infants and adults had 13 and 18%, respectively, accounting for a 2.5-fold higher frequency (**Figures 4E, H**). When merged with NK 1.1 expression, we could also observe a minor population of NKT cells across the spleen, accounting only for 0.5-1.5% of all cells (**Figures 4G, H**). However, despite their small numbers, NKT numbers in newborns were proportionally higher than in infants and adults, and even in smaller frequency, these cells displayed a progressive reduction across neonatal development (~65% reduction). Similarly, NK cells were also more frequently observed in neonates (~9%) in comparison to other ages (~4 and ~6% respectively), leading to a very similar reduction pattern across development (**Figures 4F, H**). Interestingly, anti-CD3 and anti-NK 1.1 staining revealed that these cells may form aggregates across the spleen, displayed as clusters of T, NK and NKT cells. Together, these data showed, using an *in vivo* approach, the intricate network of lymphoid cells within the spleen, that passes to major changes during postnatal development not only in cell frequency, but also in spatial organization.

Taken together, the principal component analysis (PCA; **Figure 4I**) shows that immune cell populations in 1 week old mice are sharply different from the other groups, forming very different clusters. Also, mice displayed a dispersed immune profile during the immune maturation phase. In fact, both adult and infant mice revealed high variance between individuals; however, myeloid cells seem to have similar dynamics throughout development, as do lymphoid cells, with the exception of T lymphocytes (**Figure 4J**) Therefore, our *in vivo* imaging approach revealed that, despite limitations on precise immunophenotyping, a plethora of leukocyte subtypes can be stained and imaged in their native environment, revealing novel insights on morphological and spatial aspects of spleen immune populations.

### 3.3 *In vivo* cell tracking shows an extremely dynamic network immune cells within spleen microenvironment

**Figure 5:**
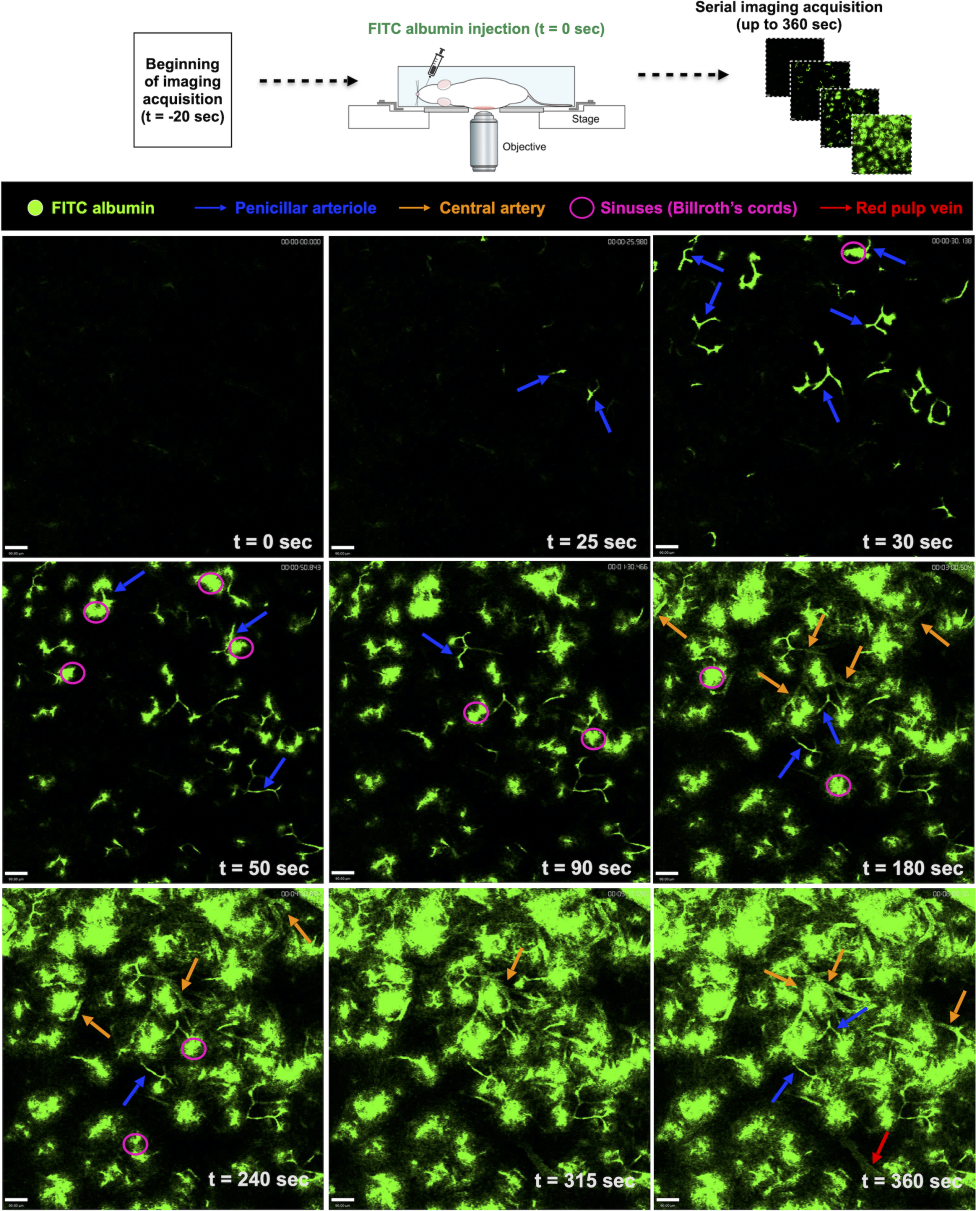
Screening for blood flow in the spleen. Snapshots of intravital movies after spleen preparation. Mice received a solution of fluorescent albumin (FITC-conjugated) intravenously, allowing the visualization of the blood flow dynamics *in vivo*. With the help of theoretical information about the direction of blood flow within the spleen, we were able to identify the splenic vascular structures.

We next used intravital microscopy to visualize the dynamics of the intricate network of immune cells within the spleen. For this, mice were anesthetized and prepared for *in vivo* imaging. However, despite a broad understanding of spleen microcirculation in histology, there is still limited data on how spleen vessels are depicted under intravital microscopy, including central arterioles, penicillar arteries, red pulp, and others. To describe and image blood dynamics *in vivo*, we prepared a solution of fluorescent albumin (FITC-conjugated) that once injected intravenously would allow a dynamic visualization of the blood flow. Then, mice were positioned in the microscope and right after movies were stated to be acquired, an *in-bolus* injection of fluorescent albumin was injected. In this way - knowing the theoretical direction and sequence of blood flow within the spleen - we were able to sequentially identify all splenic vascular structures *in vivo*. As shown in **Figure 5** and **Supplementary Movie 1**, after 25-30 seconds of endovenous FITC-albumin injection, small portions of the penicillar arteries were seen, enhancing their staining in the next timepoints. Sequentially, fluorescent plasma reached splenic sinuses (Billroth’s cords) and several fluorescent circumferential areas started to be seen across the field of view. After 180 seconds of FITC-albumin injection, larger vessels started to be visualized within the spleen, which were defined as the branches of the splenic arteries. Following a broader distribution of FITC-albumin within the vessels, areas devoid of significant staining (presumably clusters of immune cells; white pulp) were crossed by large vessels, defining the red pulp veins. Together, these data catalysed an unprecedented understanding of the *in vivo* dynamics of blood flow within the spleen under confocal intravital microscopy, paving the way for further definitions of spatial distributions of cells and structures.

**Figure 6:**
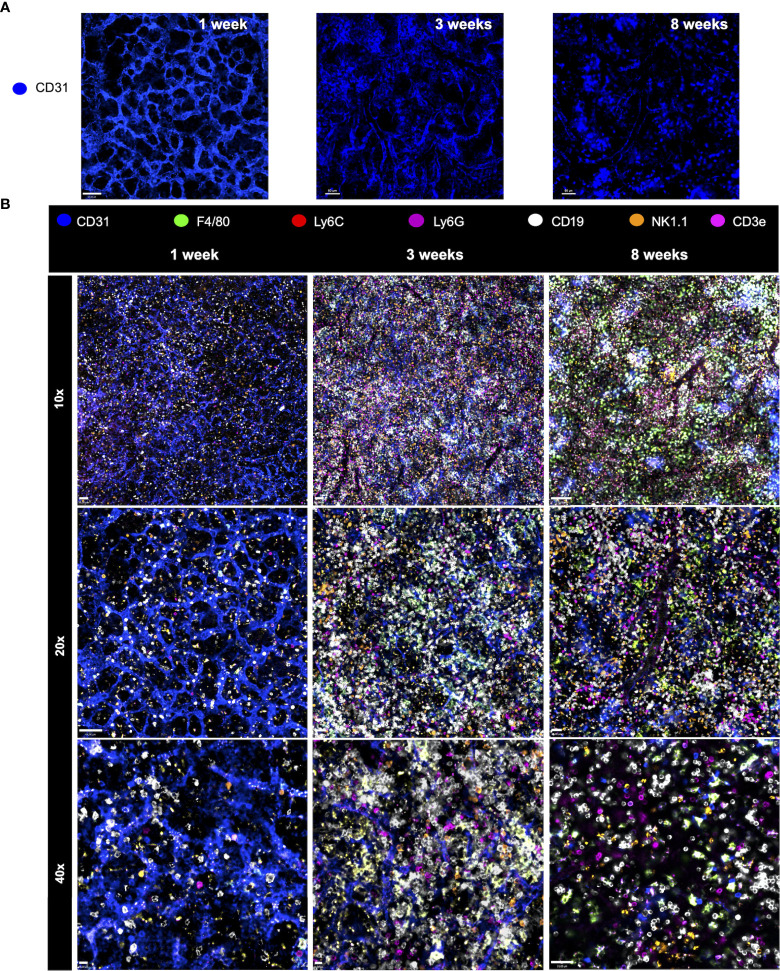
Major changes in immune cell population during the postnatal period is accompanied by alterations in the vascular network. **(A)** Changes in the spleen vascular network during postnatal development. For this, mice were injected with anti-CD31 (anti-PECAM-1; in blue; stains for endothelial cells) and imaged under confocal intravital microscopy. **(B)** Merged channels images permit the visualization of different immune cell populations at distinct ages. Spleen staining in different ages using WT mice that received i.v. anti-CD31 (in blue), anti F4-80 (in green), anti-Ly6C (in red), anti-Ly6G (in purple), anti-CD19 (in white), anti-NK1.1 (in orange) and anti-CD3e (in magenta).

We next investigated the putative changes in the spleen vascular network during postnatal development. For this, mice were injected with anti-CD31 (anti-PECAM-1; a marker for endothelial cells) and imaged under confocal intravital microscopy. As shown in **Figure 6A**, vessels in the newborn spleen display a completely different diagram in comparison to adults. Spleen vessels in neonates are extremely branched forming a web of sinusoids that resemble honeycombs. In sharp contrast, only larger vessels were visualized in adult spleens, suggesting that not only the vascular web is completely altered during development, but probably also the expression of CD31 in smaller vessels in adults. When merged with 6 channels of immune cells, as seen in **Figure 6B**, it is clear that major changes in immune cell population during the postnatal period is accompanied by alterations in vascular network.

**Figure 7:**
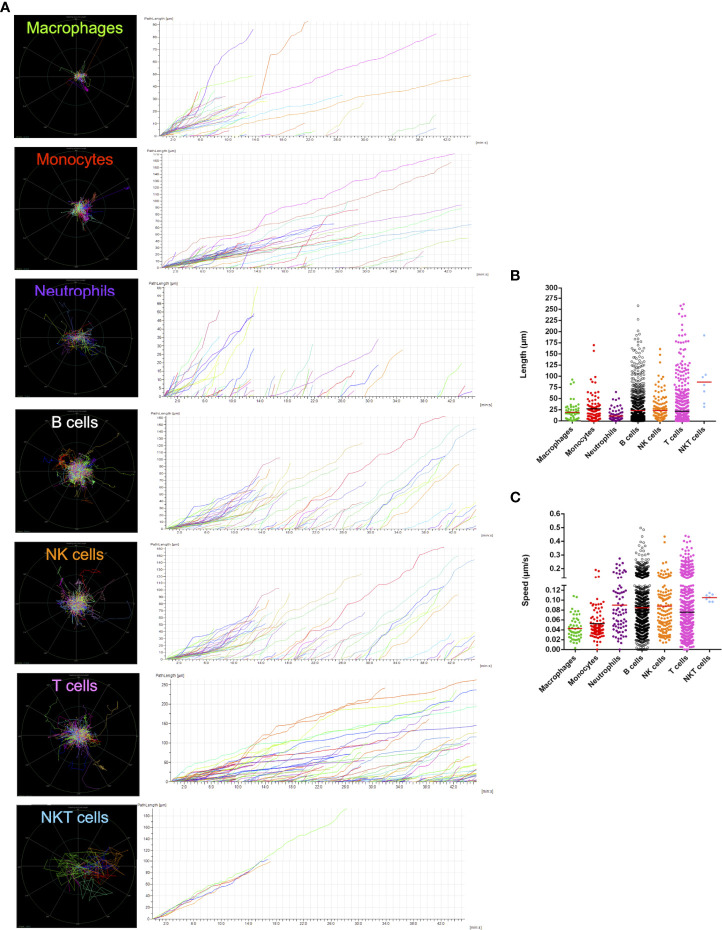
Cell tracking shows an extremely dynamic network of immune cells within the spleen microenvironment. **(A)** Digital intravital tracking of the different subtypes of leukocytes in the spleen, along with **(B)** path length and **(C)** speed information plots about each cell assessed.

Once established the guidelines for identifying both immune and endothelial cells, we next imaged spleens for longer periods to visualize the dynamics of leukocytes *in vivo*. Movies were acquired under confocal intravital microscopy, and different spleen leukocytes were digitally tracked to measure their displacement and velocity. Due to limitations to perform laparotomy and imaging for longer timepoints in newborns, only adults were studied in this protocol. As shown in **Figures 7A-C** and **Supplementary Movie 2**, spleen leukocytes are very active and motile, crawling under different moving patterns. While macrophages were mostly seen as sessile, lymphoid cells - including B, T, NK and NKT cells - displayed faster moving behaviour, accounting for longer paths in the field of view. Also, neutrophils and monocytes presented a similar movement pattern, while neutrophils were mostly found inside the microvasculature. If we consider only faster moving events, the vast majority of these were observed only in lymphoid cells, and interestingly, some of these cells had a 4-5-fold faster velocity rate, accounting for 5-10-fold longer paths within the spleen (**Figure 7**). This suggests that there might exist different populations of spleen leukocytes that are either sessile, slow- or fast-moving cells.

### 3.4 Major alterations in different subtypes of leukocytes, along with a profound reorganization of spleen immune environment, is observed during acute malaria response

**Figure 8:**
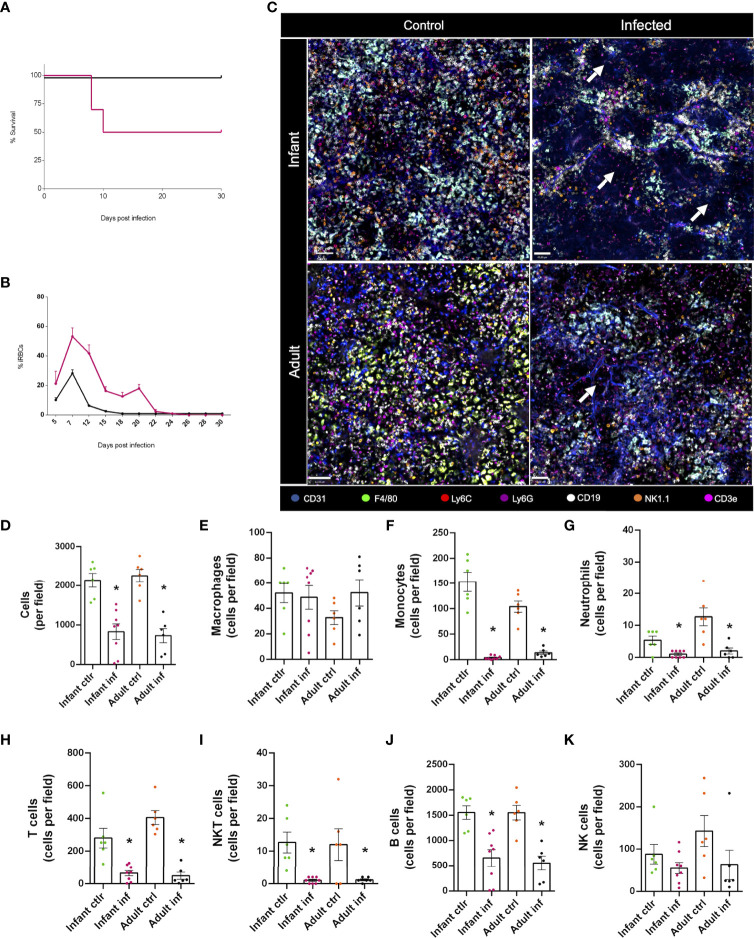
Major alterations in different subtypes of leukocytes, along with a profound reorganization of the spleen immune environment, is observed during acute malaria response. Infant (3-week-old) and adult mice (8-week-old) were infected with a murine model of malaria - *Plasmodium chabaudi* – and data was collected following disease progression. Intravenous infection with 1x10^5^ infected red blood cells (iRBCs). **(A)** Survival rate of the infected animals in both groups (infants and adults). **(B)** Percentage of iRBCs in the blood of the tested animals. **(C)** Confocal microscopy images of the spleen from both control and infected animals showing vasculature (anti-CD31, in blue), macrophages (anti F4-80, in green), monocytes (anti-Ly6C, in red), neutrophils (anti-Ly6G, in purple), B lymphocytes (anti-CD19, in white), NK cells (anti-NK1.1, in orange) and T lymphocytes (anti-CD3e, in magenta). **(D-K)** Differential expression of markers and cell morphology were analysed to quantify immune cell populations in control and infected animals, both in infant and adult groups. *p <0.05 compared to the control group. *t*-test. NK, natural killer.

In malaria-infected subjects the spleen senses subtle mechanical changes in infected and uninfected red blood cells (RBC), and by filtering the blood, this organ is responsible to regulate parasite biomass. It is well described that beyond filtering infected RBC, spleen leukocytes also mount a robust immune response to parasites once they are exposed to immune cells, and such overt inflammation is usually studied using flow cytometry, histology, and different protein/gene expression techniques. However, bioimaging approaches directed to visualize and understand immune cell dynamics, especially in their native habitat *in vivo*, are very rare in literature. To investigate such putative changes, we infected both infants and adults with a murine model of malaria - *Plasmodium chabaudi* - and followed disease progression over time. Strikingly, while adults were completely resistant to malaria infection, almost 50% of infants died upon infection (**Figure 8A**). This was accompanied by a significantly higher parasitaemia in the peak of infection, and, while adults had around 30% of infected RBC, infants peaked at more than 60% of parasitaemia (**Figure 8B**). Also, while parasites were completely absent in the circulation of adults by the 18th day post infection, infants displayed persistent infected cells until the 22nd day, with higher titers of parasitaemia. This indicated that our model of malaria induces a completely different disease profile in adults and infants, which is in complete agreement with clinical data of higher mortality and morbidity of malaria in children.

Next, we imaged spleen under multichannel confocal intravital microscopy. As shown in **Figure 8C**, *Plasmodium* infection induced a major immune cell rearrangement in the spleen of both infants and adults, depicting large areas devoid of cells. In fact, when we quantified the number of leukocytes in the field of view, we found a ~50% reduction in cellularity (**Figure 8D**), which was due to a major reduction (~90%) in the number of monocytes, neutrophils, T, NKT and B cells (**Figures 8F-J**). Macrophages (**Figure 8E**) and NK (**Figure 8K**) cells were resistant to plasmodium-induced depletion in all groups. Therefore, despite differences in mortality during acute malaria, both infants and adults displayed significant changes in the immune environment during *Plasmodium* infection, opening a new window of investigation.

## 4 Discussion

The spleen hosts all subtypes of leukocytes, including myeloid and lymphoid cells. These cells are key protectors of the organism because they identify blood borne pathogens and cellular stress, remove dying cells and foreign material, regulate tissue homeostasis and inflammatory responses, and shape adaptive immunity during postnatal development. Additionally, they can be key players in different parasitic diseases, such as bacterial and protozoan infections. Here we showed, using a unique combination of high dimensional intravital microscopy, how the splenic immune system evolves during life, describing the main immunologic changes during development after birth. Also, we paved a way to future studies that are intended to image cells under their native environment, developing not only a novel panel of seven different fluorophores that works *in vivo*, but also all the paths to use conventional confocal microscopes to work as multi-channel imaging platforms. Using this model, we also validated that our imaging setup is responsive not only to changes due to the development, but also due to major systemic infections such as malaria.

The first reports of the use of intravital microscopy date back from physiological studies after the introduction of the first fluorescence microscope by Heimstadt in 1911 (25) and after the development of exogenous fluorophores. The advent of fluorescent protein and fluorescent probes has played an important role in imaging. In 1994, with the superb advent of Green fluorescent protein (GFP) - which was originally isolated from *Aequorea victoria* - a major advance in imaging was witnessed (26). Now, combination of genetically-driven fluorescence with exogenous probes has catalysed a rapid and huge expansion on our ability to imaging living phenomena. New probes, especially those that are based on more stable polymers (i.e. Brilliant Violet dyes), allows longer exposure times for movies, and since they have more brightness efficiency, deeper tissues can be imaged and structures that were not visible using conventional fluorophores can now be revealed under high definition. Using a combination of these dyes, we could reveal previously unseen cellular organization within the spleen where several subtypes of leukocytes could be imaged at the same time. Also, regardless of limitations on antibody penetration within tissues and putative lack of staining of cells that were distant from the main vessels, we could provide enough and reliable data on the possibility of investigation of these cells, including recording movies and rendering 3D reconstructions – elevating intravital microscopy to a 11 dimensions technique (7 colours, 3 spatial direction X, Y and Z, plus time). Taken together, our data showed that spleen from newborns are sharply different from adults in almost all parameters analysed, which corroborated our previous data on liver development. We have previously shown that there is a complete shift on the immune system profile within the liver during neonatal period. Livers from newborns (0 to 14 days old mice) displayed large islands of immune cells, which were also surrounded by sinusoids with very different shapes. In these mice, classic hepatic parenchyma was almost absent (lower number of hepatocytes) and most of the liver cells were immune cells. However, in the opposite direction of adults, livers from newborns were dominated by myeloid cells, harbouring an enormous population of granulocytes. Only during the weaning period (between 2nd and 4th week of life), liver immune cells - and metabolism - reached characteristics of a mature organ (27). We observed a similar pattern with the spleen, regardless of the different immune profile across the development.

It is worth noting that there are differences between mouse and human spleens. In humans, the marginal zone lacks a clearly delimited marginal sinus and it is surrounded by an additional perifollicular zone, where some blood vessels terminate in capillaries that bypass the filtration cords as fast ‘closed’ microcirculation. Also, the mouse spleen is non-sinusoidal whereas the human spleen is sinusoidal. In in non-sinusoidal spleens, the blood flows through open-ended pulp venules provided of flat endothelium, impelling only a small impedance to entrance of red blood cells. And importantly, there is an intense erythropoiesis elicited in the mouse spleen, which remains unreported in the human spleen (28). However, even in the face of these differences, we are currently establishing that our imaging strategy is also valid for ex vivo samples and also in combination with flow cytometry (data not shown). Therefore, by selecting these combinations of seven channels, along with a strategic selection of antibodies, a plethora of cells can be imaged or immunophenotyped under high definition.

It is becoming increasingly clear that proper immune response is a cornerstone for homeostasis maintenance and prevention not only of infections, but also against other diseases such as cancer and diabetes. Amongst infectious diseases that still cause major human and financial damages worldwide, malaria remains a sad protagonist. Malaria causes substantial morbidity and mortality in many of the most resource-limited areas of the world and can rapidly develop into severe disease that can be fatal (29). Anti-parasite immune responses can efficiently control malaria parasite infection at all development stages, and under certain circumstances they can even prevent parasite infection. However, translating these findings into vaccines or immunotherapeutic interventions has been difficult in part because of the high biological complexity of this parasite, and this is worse when considering children, since it is estimated that every 75 seconds, a child under five dies of malaria. Considering the relevance of immune response to control malaria infection, the major participation of spleen in its pathology and the major differences of immune response in children in comparison to adults, our data can shed some light in future investigations on how pharmacological interventions and therapies could be more specific and directed to different ages.

In summary, we described a novel combination of selected antibodies and fluorophores to image *in vivo*, for the first time, the spleen immune environment during all phases of development. Also, we revealed how malaria can change the spleen immune profile in adults and infants, which could become the key to understanding different severity grades of infection. Our new imaging solutions can be extremely useful for different groups in all areas of biological investigation, paving a way for new intravital approaches and advances.

## Data availability statement

The original contributions presented in the study are included in the article/**Supplementary Material**. Further inquiries can be directed to the corresponding author.

## Ethics statement

The animal study was reviewed and approved by CEUA - UFMG.

## Author contributions

Conceptualization and investigation: ML, RB, RL, and GM. Experiments, sample collection, data discussion: MP-P, CM, ML, BN, KM, CP, KC, AD, MA, and GM. Data analysis: MP-P, AO and GM. Writing the original draft: MP-P and GM. Supervision and project administration: GM. All authors contributed to the article and approved the submitted version.

## Funding

This work was supported by FAPEMIG, CAPES, CNPq (Brazil), INCT Vacinas and Chang Zuckerberg Initiative (Bioimaging Brasil).

## Acknowledgments

We thank BD Biosciences and Nikon for technical support. Also, we thank Dr. Ricardo Gazzinelli for support with malaria experiments.

## Conflict of interest

The authors declare that the research was conducted in the absence of any commercial or financial relationships that could be construed as a potential conflict of interest.

## Publisher’s note

All claims expressed in this article are solely those of the authors and do not necessarily represent those of their affiliated organizations, or those of the publisher, the editors and the reviewers. Any product that may be evaluated in this article, or claim that may be made by its manufacturer, is not guaranteed or endorsed by the publisher.

## Supplementary material

Supplementary Video 1 Blood flow examination through fluorescent albumin (FITC-conjugated) staining. Autofluorescence (blue); FITC-albumin (green).

Supplementary Video 2 Immune cells in splenic microenvironment from an 8-week mouse. Endothelial cells (anti-CD31, in blue), macrophages (anti F4-80, in green), monocytes (anti-Ly6C, in red), neutrophils (anti-Ly6G, in purple), B lymphocytes (anti-CD19, in white), NK cells (anti-NK1.1, in orange) and T lymphocytes (anti-CD3e, in magenta).

Supplementary Video 3 3D video of the spleen from a 1-week mouse. Media shows vasculature (anti-CD31, in blue), macrophages (anti F4-80, in green), monocytes (anti-Ly6C, in red), neutrophils (anti-Ly6G, in yellow), B lymphocytes (anti-CD19, in white), NK cells (anti-NK1.1, in orange) and T lymphocytes (anti-CD3e, in magenta).

Supplementary Video 4 3D video of the spleen from a 3-week mouse. Media shows vasculature (anti-CD31, in blue), macrophages (anti F4-80, in green), monocytes (anti-Ly6C, in red), neutrophils (anti-Ly6G, in yellow), B lymphocytes (anti-CD19, in white), NK cells (anti-NK1.1, in orange) and T lymphocytes (anti-CD3e, in magenta).

Supplementary Video 5 3D video of the spleen from an 8-week mouse. Media shows macrophages (anti F4-80, in green), monocytes (anti-Ly6C, in red), neutrophils (anti-Ly6G, in yellow), B lymphocytes (anti-CD19, in white), NK cells (anti-NK1.1, in orange) and T lymphocytes (anti-CD3e, in magenta).
